# Chest X-Ray Images to Differentiate COVID-19 from Pneumonia with Artificial Intelligence Techniques

**DOI:** 10.1155/2022/5318447

**Published:** 2022-12-22

**Authors:** Rumana Islam, Mohammed Tarique

**Affiliations:** ^1^Department of ECE, University of Windsor, ON, Canada N9B 3P4; ^2^Department of ECE, University of Science and Technology of Fujairah, UAE

## Abstract

This paper presents an automated and noninvasive technique to discriminate COVID-19 patients from pneumonia patients using chest X-ray images and artificial intelligence. The reverse transcription-polymerase chain reaction (RT-PCR) test is commonly administered to detect COVID-19. However, the RT-PCR test necessitates person-to-person contact to administer, requires variable time to produce results, and is expensive. Moreover, this test is still unreachable to the significant global population. The chest X-ray images can play an important role here as the X-ray machines are commonly available at any healthcare facility. However, the chest X-ray images of COVID-19 and viral pneumonia patients are very similar and often lead to misdiagnosis subjectively. This investigation has employed two algorithms to solve this problem objectively. One algorithm uses lower-dimension encoded features extracted from the X-ray images and applies them to the machine learning algorithms for final classification. The other algorithm relies on the inbuilt feature extractor network to extract features from the X-ray images and classifies them with a pretrained deep neural network VGG16. The simulation results show that the proposed two algorithms can extricate COVID-19 patients from pneumonia with the best accuracy of 100% and 98.1%, employing VGG16 and the machine learning algorithm, respectively. The performances of these two algorithms have also been collated with those of other existing state-of-the-art methods.

## 1. Introduction

On March 11, 2020, the World Health Organization (WHO) declared the COVID-19 outbreak a pandemic [1]. Initially, this unique virus emerged in Wuhan, China, and was named a novel coronavirus. Later, the International Committee on Taxonomy of Viruses renamed this virus as severe acute respiratory syndrome coronavirus 2 (SARS-CoV-2). Since then, millions of people worldwide have been infected by this coronavirus and its variants [2].

To prevent the spreading of this virus, control mechanisms, including wearing facemasks and massive testing campaigns, have been suggested [3]. Wearing masks have been mandated in public places by many government and private organizations worldwide. Even a convolutional neural network (CNN) based facemask detection algorithm has been developed by researchers [4] to enforce wearing masks in public places.

The reverse transcription-polymerase chain reaction (RT-PCR) has been introduced to test the coronavirus and is still considered the gold standard for testing this virus [5]. However, there are some limitations of the RT-PCR test: (a) it is not economical, (b) it needs variable time to produce the results, (c) it necessitates person-to-person contact to administer, and (d) it is not even reachable to the major population due to a lack of healthcare facilities [6]. Moreover, the RT-PCR test is invasive and uncomfortable for patients, specifically children. To overcome these limitations, researchers strived to find an alternative to the RT-PCR test, and they have recommended using noninvasive techniques instead. Biomedical signals and radiological images are recommended for this purpose.

Biomedical signals, including speech, vowels, words, phrases, and counting numbers, have been used to detect several diseases [7–9]. Literature survey shows that these signals can be used to detect various diseases, including asthma [10], Alzheimer's disease [11], Parkinson's disease [12], vocal fold diseases [13], depression [14], schizophrenia [15, 16], autism [17, 18], dysphonia [19], abnormality in fetal heart rate [20], and breast cancer [21]. Recent works have also demonstrated that coughing sounds could detect respiratory disorders in COVID-19 patients [22, 23]. However, biomedical signal-based disease diagnosis requires sophisticated equipment to administer. In addition, only trained technologists can perform the signal acquisition, processing, and analysis tasks. Biomedical images can overcome these limitations.

Biomedical images have been used for diagnosing diseases in plants [24] and animals for a long time. The three significant steps followed by these diagnoses are (a) preprocessing, (b) image feature extraction, and (c) classification. The preprocessing techniques may include image acquisition, image resizing, image enhancement, image segmentation, and extracting the region of interest (ROI). Then, image features are extracted from the preprocessed images. Finally, these features are applied to a classifier for final diagnosis.

Recently, deep learning-based algorithms are playing an essential role as classifiers. For example, an enhanced deep learning-based CNN model with a leaky rectified linear unit (ReLU) activation function has been proposed in [25] to detect a skin disease called acne. The authors have used different image processing techniques in their work, namely, *k*-means, texture analysis, and segmentation. The results show that the deep learning-based algorithm can achieve a higher accuracy (i.e., 97.54%) than the SVM algorithm while detecting this disease.

**Figure 1:**
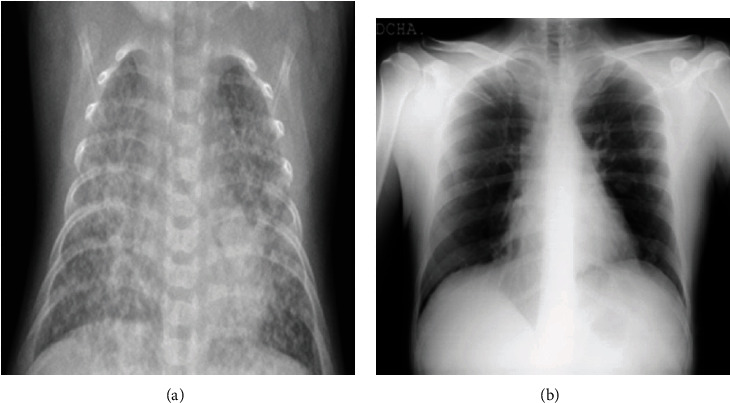
The sample X-ray images of (a) a viral pneumonia patient and (b) a COVID-19 patient as collected from the Kaggle database [46].

Biomedical images, including computerized tomography (CT) and X-ray images, are popularly used to diagnose lung diseases like pneumonia [26–29], tuberculosis [30–32], interstitial lung diseases [33], early lung cancer [34–37], and pulmonary nodules [38–43]. The key advantages of using radiological images are the following: (a) they can be readily produced at any medical facility equipped with the necessary instrument and (b) the physicians need considerably less time to perform visual subjective diagnoses. The chest CT image is generally computed by scanning techniques, whereas the X-ray images are captured from different angles and compiled to form a single image. The CT scans provide physicians with more detailed information about the patient for diagnoses compared to the X-ray images. However, CT scans are more expensive than their X-ray counterparts and are available only in specialized healthcare facilities. This investigation considers the chest X-ray images only. However, comparing the chest X-ray images of patients with COVID-19 and other lung diseases often leads to wrong diagnoses [44, 45]. For example, it is hard to subjectively differentiate between the X-ray images of a COVID-19 patient and a viral pneumonia patient, as shown in Figure 1. However, early discrimination and isolation of COVID-19 patients from pneumonia patients are vital to prevent the pandemic's spreading. It is also essential for healthcare facilities to reduce their ever-increasing burden.

This work presents a noninvasive technique to detect COVID-19 patients from chest X-ray images and artificial intelligence. A deep CNN and several machine learning algorithms have been used as classifiers. The CNN is trained with original chest X-ray images. On the other hand, the machine learning algorithms are matriculated with encoded image features extracted from the chest X-ray images. The main contributions of this work are as follows:

- To develop a classification model to differentiate COVID-19 and pneumonia patients using lung X-ray images based on machine learning and deep learning approaches
- To extract encoded image features and investigate their usefulness in identifying COVID-19 and pneumonia patients using several machine learning algorithms
- To reduce the computational burden and hence to faster the algorithm, using a data reduction technique
- To provide a detailed performance analysis of the proposed classification systems in terms of statistical performance parameters
- To compare the performances of the proposed techniques with those of other state-of-the-art algorithms

The rest of the paper is organized as follows. Related works are presented in Section 2. Materials and methods are presented in Section 3. Simulation results are presented in Section 4. The paper is concluded with Section 5. A list of acronyms used throughout this paper is provided in Table 1.

## 2. Related Works

Recently, COVID-19 patient detection, using X-ray images and artificial intelligence, has drawn considerable attention from researchers. One of the earliest works can be found in [47]. The authors have used seven different architectures of CNN to detect COVID-19 patients. They achieved the best detection accuracy with the VGG19 (90%) and DenseNet (90%).

Two deep learning models, VGG19 and U-Net, have been deployed in [48] to process the X-ray images and classify them as COVID-19 positive or COVID-19 negative. The proposed system preprocessed the images by segmentation and then categorized them using a transfer learning scheme. The authors achieved a detection accuracy of around 97%.

An Android application was designed in [49] to identify COVID-19 patients using X-ray images. For this purpose, a CNN was developed and deployed on an Android mobile phone. By employing a 5-fold cross-validation, the authors achieved an average accuracy, sensitivity, specificity, precision, and F1-score of 98.65%, 98.49%, 98.82%, 98.81%, and 98.65%, respectively.

To overcome the limitation imposed by the small dataset, a deep convolutional generative adversarial network (DCGAN) has been used in [50]. The DCGAN regenerates enough data from the limited existing data for the training task and hence overcomes the constraint of a limited dataset. The simulation showed that the DCGAN could successfully classify the X-ray images into normal, pneumonia, and COVID-19.

Supervised machine learning techniques have been used in [51] to detect COVID-19 patients based on X-ray images. The authors have extracted a color layout descriptor (CLD) feature from the images. The results show that the CLD can assist a machine learning algorithm in achieving a high precision and recall value while discriminating COVID-19 from other pulmonary diseases.

A novel machine learning algorithm called the Siamese CNN model was proposed in [52] to detect COVID-19 automatically by utilizing X-ray images. The authors have used three consecutive models in parallel to extract the image features. The results showed that the proposed algorithm achieved an accuracy of 96.70% while classifying the X-ray images into COVID-19, non-COVID-19, and pneumonia. In a similar work [53], common bacterial pneumonia, COVID-19, and healthy subjects have also been investigated. The authors have used a transfer learning scheme in their work. They achieved the accuracy, sensitivity, and specificity of 96.78%, 98.66%, and 96.46%, respectively.

In [54], the authors have introduced a novel network architecture to detect COVID-19. They replaced the final classifier layer in the DenseNet-201 with a new network consisting of a global averaging layer, a batch normalization layer, a dense layer with ReLU activation, and a final classification layer. They achieved an accuracy of 94% while detecting COVID-19.

A pretrained Res-CovNet has been used in [55] to classify the X-ray images of healthy, bacterial pneumonia, viral pneumonia, and COVID-19. The authors have introduced a novel framework using the Internet of Medical Things (IoMT) to collect X-ray images from remotely located patients. The results showed that the proposed model could discriminate COVID-19 patients from healthy patients with an accuracy of 98.4%. However, the proposed model detected COVID-19 patients from normal, bacterial pneumonia, and viral pneumonia with a lower accuracy (i.e., 96.2%).

Fifteen (15) pretrained CNN models have been used in [56]. The authors achieved the highest accuracy with the VGG19.

Computer vision algorithms and medical image analysis techniques have been used in [57] to identify COVID-19. For this purpose, the authors have employed three state-of-the-art deep learning models, namely, ResNet-V2, InceptionNet-V3, and NASNetLarge. They have investigated two techniques, namely, (a) with data augmentation and (b) without data augmentation. They achieved 98.63% and 99.02% accuracies for these two cases, respectively.

A pretrained novel network model called ResNet-50 and several image processing techniques, including augmenting, enhancing, normalizing, and resizing, have been used in [58] to detect COVID-19 patients. The results showed that the proposed system outperformed other algorithms, including VGG16, VGG19, and DenseNet.

In [59], the Apache Spark system has been utilized as an extensive data framework to collect the X-ray images of healthy and COVID-19 subjects. Three models, namely, Inception-V3, TestNet-50, and VGG19, have been investigated in their work. All these three models achieved an accuracy of 100% while discriminating COVID-19 samples from healthy samples.

A large dataset of X-ray images has been investigated in [60]. In this investigation, the authors used the X-ray images of the COVID-19 patients from Github and the healthy X-ray images from the Kaggle website. The authors achieved an accuracy of 100%, and the credit went to this large dataset.

A persuasive classification and reliable detection of the COVID-19 algorithm have been presented in [61]. The authors have used the existing state-of-the-art CNN algorithms in their work. They also built a novel CNN from scratch in this work. The achieved accuracy was 100% for COVID-19 and healthy classifications. A classification accuracy of 93.75% was achieved to categorize healthy, COVID-19, and pneumonia patients.

In [62], the authors have used three features, namely, hand-crafted features, radionics features (specialized for medical images), and deep features (extracted by a pretrained deep learning architecture). The authors have combined these features and made shallow hand-crafted features. They concluded that these shallow features performed better than those individual feature sets. Four models, namely, Inception-V3, MobileNet, Xception, and DenseNet, have been used in [63] to detect COVID-19 patients using X-ray images. Based on the performance parameters for accuracy, recall, and F1-score, the authors recommend using MobileNet to detect COVID-19 patients.

CT and radiographic images (chest X-rays) have been used in [64] to detect COVID-19. The authors have used two deep learning algorithms for classification, namely, VGG19 and ResNet-50. The simulation results showed that the X-ray images have higher accuracy than the CT images.

Nine deep learning algorithms, namely, MobileNet-V2, ResNet-50, Inception-V3, NASNet-Mobile, VGG16, Xception, Inception, ResNet-V2, and DenseNet-121, have been used in [65] to detect COVID-19 patients. The authors recommended using these pretrained deep learning models as they are very fast to produce results compared to the RT-PCR test. In [66], the authors have used data processing techniques, including dataset balancing, medical experts' image analysis, and data augmentation, to implement their algorithm. They achieved an accuracy of 99%.

Unlike other works mentioned above, both chest X-ray images and symptoms have been considered in [67] to detect COVID-19. The symptoms included cough, fever, sore throat, headache, and shortness of breath. These symptoms were preprocessed and applied to a logistic regression analyzer to diagnose COVID-19 patients. Similarly, the chest X-ray images were preprocessed to classify the samples as normal, non-COVID-19-viral, bacterial, and COVID-19 positive. A decision tree algorithm combined the results of logistic regression and multiclass classification for the final classification. The proposed algorithm achieved an accuracy of 78.88%, a specificity of 94%, and a sensitivity of 77%.

Both deep learning and machine learning algorithms have been used in [68] to detect COVID-19 using chest X-ray images. The authors conducted 38 experiments using CNN, 10 experiments using five machine learning algorithms, and 14 experiments using state-of-the-art pretrained networks. They achieved a mean sensitivity, specificity, accuracy, and area under the curve (AUC) of 93.84%, 99.18%, 98.50%, and 96.51%, respectively.

In addition to the hyperparameter tuning, multiobjective adaptive differential evolution (MADE) has been introduced in [69] to detect COVID-19 using a CNN. The simulation results showed that the proposed algorithm achieved an accuracy of 94.48%, which is higher than that of other machine learning algorithms, including random forest (RF), CNN-SVM, DarkNet-19, reduced support vector machine (RSVM), DarkCOVIDNet, DeTrack, and deep transfer learning (DTL).

The abovementioned related works have used different approaches and techniques to detect COVID-19 and achieved varying accuracy levels. However, these works have some limitations too. The major limitations of the abovementioned related works are as follows.

- The algorithms need a huge dataset to train the classifiers
- The computational complexity of the algorithms is very high as there are considerable parameters to deal with
- Most of the algorithms mainly apply image processing techniques that are also computationally expensive
- The accuracy of the algorithms is still not very high (in general), although a couple of algorithms achieved an accuracy of 100%

## 3. Materials and Methods

### 3.1. Database

The X-ray images available on the Kaggle website [46] have been used in this investigation. This database is created to assist scientists, clinicians, and healthcare experts in COVID-19 diagnosis. It is one of the most popular databases used by the scientific community. These X-ray images are collected from different sources and stored in the Kaggle database. This database contains the chest X-ray images of 3616 COVID-19-positive cases, 10192 normal (healthy) cases, 6012 lung opacity (non-COVID-19 lung infection) cases, and 1345 viral pneumonia cases. This investigation uses 280 X-ray images of viral pneumonia and COVID-19 samples. These images are randomly selected from the Kaggle database. Seventy (70%) percent of these X-ray images are used for training. The remaining 30% are equally divided for validation and testing purposes.

### 3.2. Classification Algorithms

**Figure 2:**
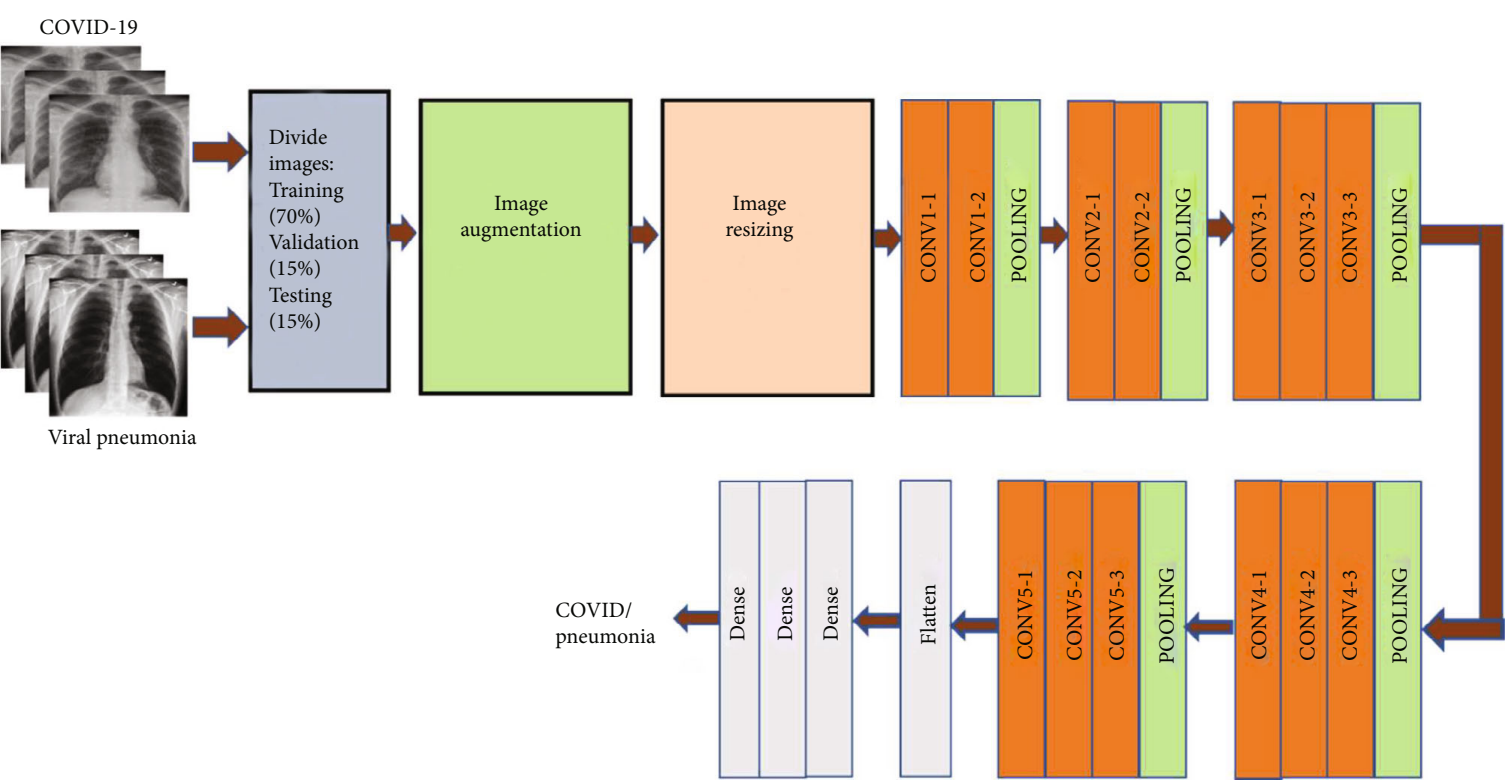
The configuration of the VGG16-based algorithm.

**Figure 3:**
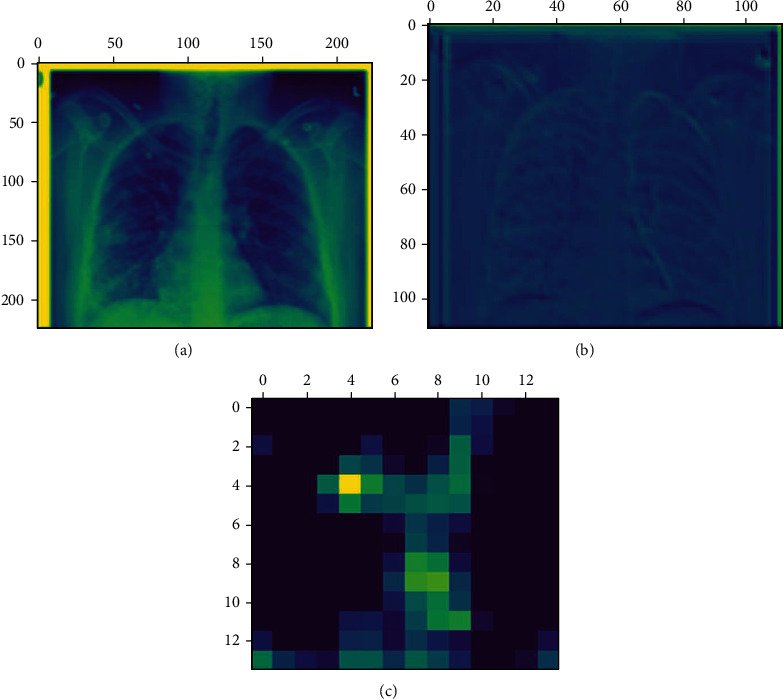
The dimension reduction technique as visualized in VGG16 networks: (a) the 24^th^ channel of the activation for the 1^st^ layer of the COVID-19 X-ray image, (b) the 1^st^ channel of the activation for the 5^th^ layer of the COVID-19 X-ray image, and (c) the 1^st^ channel of the activation for the 17^th^ layer of the COVID-19 X-ray image.

In this work, CNN and seven machine learning algorithms have been used for COVID-19 detection. CNN has been popularly used in image classification tasks. The unique characteristic of CNN is that it can recognize patterns in an image irrespective of its orientation [70]. However, CNN requires a large dataset for the training. On the other hand, the publicly available databases, including Kaggle, provide a limited dataset. Hence, a transfer learning approach has been used in this work. A pretrained network model called VGG16 has been used for this investigation. The VGG16 network is already trained on large datasets (i.e., 22000 categories of images) and is also available as prepackaged with the Keras. The VGG16 used in this work consists of a stack of 13 convolutional layers followed by three fully connected layers, as shown in Figure 2. All hidden layers use the ReLU activation function, and the final layer uses the Softmax activation function. The original X-ray images are rescaled to a fixed size of 224 × 224. Five max-pooling layers carry out spatial pooling to reduce the dimension of the data. This kind of dimension reduction technique is illustrated in Figure 3. As demonstrated in this figure, the image features vary at the different network levels and become more abstract as the layer increases. The detailed steps used by the VGG16 are illustrated in Algorithm 1. The network model was implemented in Google Colab [71].

The investigated 7 machine learning algorithms are available with the Statistics and Machine Learning Toolbox of MATLAB 2020. These seven algorithms were selected among the available machine learning algorithms as they provided the best accuracies. The chosen machine learning algorithms are (a) linear discriminant analysis (LDA), (b) fine tree, (c) logistic regression, (d) coarse Gaussian support vector machine, (e) cosine *k*-nearest neighbors (kNN), (f) ensemble subspace discriminant, and (g) linear SVM.

These machine learning algorithms use the speeded-up robust features (SURF) [72–75] extracted from the X-ray images. The SURF is a fast and robust approach that has been popularly used in implementing computer vision algorithms [76–80]. It is computed by using two main steps, namely, (a) feature extraction and (b) feature description. The feature extraction step consists of three stages, namely, (a) integral image formation, (b) Hessian matrix-based interest point detection, and (c) scale-space formation. The integral image at location **x** = (*x*, *y*)^*T*^ represents the sum of all pixels in the input image *I* within a rectangular image formed by

(1) $$\begin{array}{c} I_{\text{sum}}\left(x\right)=\overset{i\leq x}{\underset{i=0}{\sum }}\overset{i\leq y}{\underset{j=0}{\sum }}I\left(i,j\right). \end{array}$$

The integral image formation expedites the computation of the SURF feature. The SURF feature then uses the Hessian matrix to find the interest points in the integral image. The Hessian matrix *H*(*x*, *σ*) in *x* at scale *σ* is defined as

(2) $$\begin{array}{c} H\left(x,\sigma \right)=\left[\begin{array}{cc} L_{xx}\left(x,\sigma \right) & L_{xy}\left(x,\sigma \right)\\ L_{xy}\left(x,\sigma \right) & L_{yy}\left(x,\sigma \right) \end{array}\right], \end{array}$$

where *L*_*xx*_(*x*, *σ*) is the convolution of the Gaussian second-order derivative with the image *I* at point *x* in the *x*-direction. Similarly, *L*_*xy*_(*x*, *σ*) is the convolution of the Gaussian second-order derivative with the image *I* at point *x* in the *x*-direction and *y*-direction, and *L*_*yy*_(*x*, *σ*) is the convolution of the Gaussian second-order derivative with an image *I* in the *y*-direction.

To calculate the determinant of the Hessian matrix, first, the convolution is applied with the Gaussian kernel, and then, the second-order derivative is calculated. To reduce the computational cost, the SURF uses approximation approaches using box filters to compute convolution and second-order derivatives. In this work, a box filter of size 8 × 8 is used. Denoting the approximation of the Hessian defined by *D*_*xx*_, *D*_*xy*_, and *D*_*yy*_, the derivative of *H*(*x*, *σ*) can be approximated by

(3) $$\begin{array}{c} \det\left(H_{\text{approx}}\right)=D_{xx}D_{yy}-{\left(0.9D_{xy}\right)}^{2}. \end{array}$$

Then, the scale spaces are implemented by image pyramids. The images are repeatedly smoothed with a Gaussian function and are subsampled to achieve a higher-level representation in the image pyramid. The scale space is analyzed by upscaling the filter size. By increasing the filter size and doubling the sampling intervals for the interest point extraction, the upscaling of the filter is accomplished at a constant rate.

**Figure 4:**
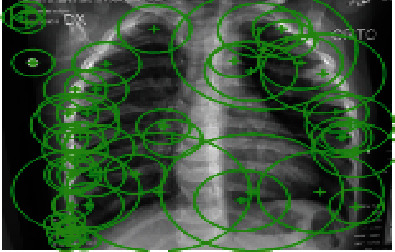
The discriminative SURF features extracted from the X-ray image.

The creation of the SURF descriptor takes place in two steps, namely, (a) orientation assignment and (b) descriptor extraction. By using the orientation assignment, the SURF is made rotation invariant. To achieve this, the SURF feature calculates the Haar wavelet response in the *x*-direction and *y*-direction. This is done in a circular neighborhood of radius 6*σ* and around the key points, where *σ* is the scale at which the key points are detected, as shown in Figure 4. Then, the sum of vertical and horizontal wavelet responses in a scanning area is calculated.

**Figure 5:**
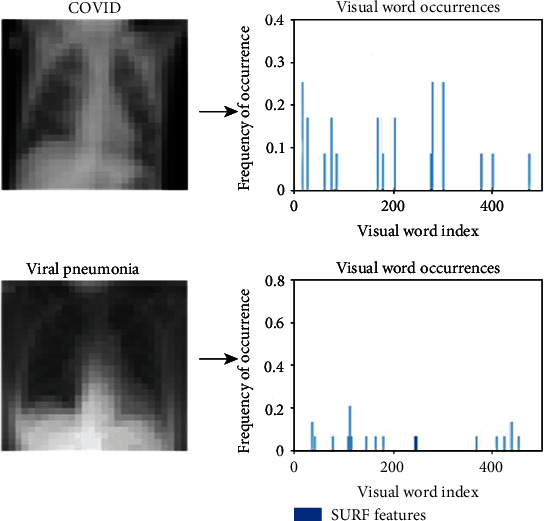
The visual encoded features of the X-ray images for COVID-19 and pneumonia patients.

**Figure 6:**
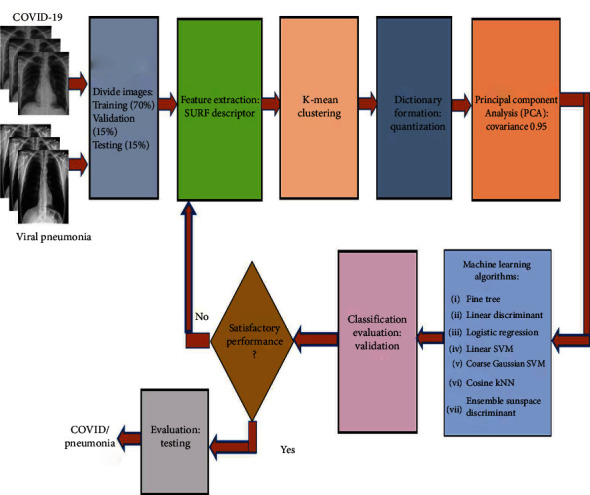
The system model employing the machine learning algorithms.

To extract the descriptor, the first step consists of constructing a square region centered around the key points and oriented along with the orientation mentioned above. Then, the region is split into smaller 4 × 4 square subregions. A few features are computed for each subregion at regularly spaced sample points. Assuming that *H*(*x*) is the Haar wavelet response in the horizontal direction and *H*(*y*) is the Haar wavelet response in the vertical direction, then *H*(*x*) and *H*(*y*) are weighted by a Gaussian centered at the key point. The wavelet responses *H*(*x*) and *H*(*y*) are summed up over each subregions, and a feature vector is formed. To cope with the intensity changes, the absolute sum of the *H*(*x*) and *H*(*y*) is also calculated. Hence, each subregion has a four-dimensional descriptor vector, *V* = (*H*(*x*), *H*(*y*), |*H*(*x*)|, |*H*(*y*)| ). This results in a descriptor vector for all 4 × 4 subregions of length 64. To further reduce the computation cost, eighty percent (80%) of the most important features were selected from the X-ray images, and the rest of them were discarded. Then, a 500-word visual vocabulary is formed by using a *k*-means clustering algorithm. The encoded visual word occurrences for the X-ray images of a COVID-19 patient and a pneumonia patient are shown in Figure 5. This figure demonstrates that the visual word occurrences for COVID-19 and pneumonia patients are distinctly different. Finally, the feature vectors are formed for the chest X-ray images, and the dimensions are reduced further by principal component analysis (PCA) with a covariance of 0.95. The computation of the steps mentioned above is illustrated in Figure 6. Once the feature vectors are formed, they are applied to the machine learning algorithms for classification. The detailed steps for the classification of the X-ray images by using machine learning algorithms are illustrated in Algorithm 2.

## 4. Results and Discussion

As stated earlier, the proposed algorithm discriminates the X-ray images of COVID-19 patients from pneumonia patients. The performances of the proposed system are evaluated with the commonly accepted measures of accuracy, precision, recall/sensitivity, and F1-score as described in the following equations [81, 82]. The evaluation parameters used in the equations are as follows: (a) TP (true positive): the X-ray image belongs to COVID-19, and the algorithm correctly diagnoses it as COVID-19; (b) TN (true negative): the X-ray image belongs to pneumonia, and the algorithm correctly evaluated it as pneumonia; (c) FP (false positive): the X-ray image belongs to pneumonia, but the algorithm wrongly diagnosed it as COVID-19; and (d) FN (false negative): the X-ray image belongs to COVID-19, but the algorithm wrongly diagnosed it as pneumonia. The performance measures investigated are defined as follows.

Accuracy is the ratio of the correctly predicted observations to the total observations. It is defined by

(4) $$\begin{array}{c} \text{Accuracy}=\frac{\text{TP}+\text{TN}}{\text{TP}+\text{FP}+\text{FN}+\text{TN}}. \end{array}$$

Precision/positive predictive value (PPV) is the ratio of correctly predicted positive observations to the total predicted positive observations. It is defined by

(5) $$\begin{array}{c} \text{Precision}/\text{PPV}=\frac{\text{TP}}{\text{TP}+\text{FP}}. \end{array}$$

Recall/true positive rate (TPR) is the ratio of correctly predicted positive observations to all observations in the actual class. It is defined by

(6) $$\begin{array}{c} \text{Recall}/\text{TPR}=\frac{\text{TP}}{\text{TP}+\text{FN}}. \end{array}$$

The false detection rate (FDR) is the expected ratio of false positive observations to the total number of positive observations. It is defined by

(7) $$\begin{array}{c} \text{FDR}=\frac{\text{FP}}{\text{FP}+\text{TP}}. \end{array}$$

A false negative rate (FNR) is the test's probability of missing a true positive. It is defined as

(8) $$\begin{array}{c} \text{FNR}=\frac{\text{FN}}{\text{FN}+\text{TP}}. \end{array}$$

F1-score is the weighted average of precision and recall. Therefore, this score takes both false positives and false negatives into account. F1-score is defined by

(9) $$\begin{array}{c} \text{F}1‐\text{score}=\frac{2*\text{Recall}*\text{Precision}}{\text{Recall}+\text{Precision}}. \end{array}$$

The geometric mean, *G*-mean, reveals the contribution of sensitivity and specificity. It is formulated as

(10) $$\begin{array}{c} G‐\text{mean}=\sqrt{\text{Sensitivity}*\text{Specificity}}. \end{array}$$

Matthew's correlation coefficient considers all evaluation parameters into account, as defined by

(11) $$\begin{array}{c} \text{ MCC}=\frac{\text{TP}*\text{TN}-\text{FP}*\text{FN}}{\sqrt{\left(\text{TP}+\text{FP}\right)\left(\text{TP}+\text{FN}\right)\left(\text{TN}+\text{FP}\right)\left(\text{TN}+\text{FN}\right)}}. \end{array}$$

**Figure 7:**
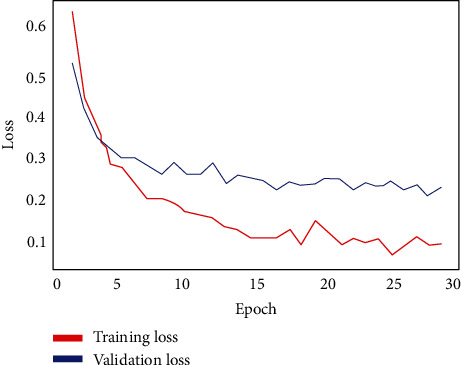
The training and validation losses for VGG16.

**Figure 8:**
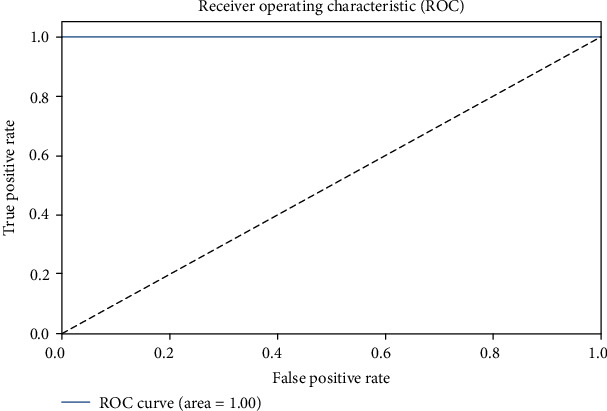
The ROC characteristics of VGG16.

The performance of the system model with VGG16 was optimized with the parameters listed in Table 2. The training and validation losses are plotted in Figure 7. The performance metrics are summarized in Table 3. It shows that the system model with VGG16 achieves the optimum values for the precision, recall, F1-score, *G*-mean, and MCC all equal to 1.0. The table also shows the log loss score of 0.0373 only. The receiver operating characteristic (ROC) is shown in Figure 8. The ROC demonstrates that the AUC is 1.0, indicating that the VGG16 could correctly distinguish the X-ray images of the COVID-19 patients from pneumonia patients with an accuracy of 100%.

The simulations were repeated with the encoded SURF features employing seven top-performing machine learning algorithms as mentioned in the previous section. The performances of these machine learning algorithms are listed in Tables 4 and 5. These tables list the performances in terms of TPR, FNR, PPV, FDR, and F1-score.  Table 4 shows that the coarse Gaussian SVM provides the highest TPR of 98.8%, the lowest FNR of 1.2%, and the highest F1-score of 98.15% while detecting COVID-19 patients. The linear SVM provides the highest PPV of 98.70% and the lowest FDR of 1.3%.

Table 5 presents the performances of the machine learning algorithms for detecting pneumonia. This table shows that the highest TPR of 98.8% is achieved by the linear SVM and cosine kNN algorithms. The linear SVM also provided the highest F1-score of 98.14%. The coarse Gaussian SVM algorithm provides the highest PPV of 98.70%. The performance comparison of the machine learning models is presented in Table 6, which shows that the linear SVM and coarse Gaussian SVM provide the highest accuracy of 98.10%. But the coarse Gaussian SVM demonstrates the fastest prediction rate (2.04 ms/prediction). Also, the corresponding AUCs are displayed for each machine learning algorithm. Based on the simulation results presented in Tables 4–6, it can be concluded that the SVM provides the best performance in terms of accuracy, prediction rate, and AUC.

Finally, the proposed method's performances are compared with those of the recently published works. The comparison results are listed in Table 7. This table shows that the highest accuracy of 100% was achieved with the pretrained VGG16 which is comparable to the works presented in [59–61, 63], as listed in Table 7. This table also shows that two machine learning algorithms, linear SVM and coarse Gaussian SVM, also achieve an accuracy of 98.1% with the SURF features. This accuracy is higher than that of the algorithms presented in [47, 48, 52–54, 56, 62, 65, 67] in Table 7. Although the accuracy is a little less than that achieved with the VGG16, considering the lowest training and prediction rate (09.72 seconds and 2.04 milliseconds), the system justifies its viability with the SURF feature to differentiate COVID-19 patients from pneumonia using chest X-ray images with the machine learning algorithms.

## 5. Conclusion

This paper presented a noninvasive, automated detection algorithm to identify COVID-19 patients from viral pneumonia patients based on the X-ray images objectively. A deep pretrained CNN model, VGG16, and several machine learning algorithms were investigated. Despite the visual similarities between the X-ray images of pneumonia and COVID-19 patients, it is shown that wisely selected machine learning algorithms and extraction of discriminative features from the images could successfully discriminate them. Among the investigated machine learning algorithms, the SVM was able to differentiate the X-ray images of COVID-19 patients from viral pneumonia patients with the highest accuracy of 98.1%. It was also shown that the deep pretrained VGG16 achieved an accuracy of 100% even with the limited data samples.

In the future, other pulmonary diseases like asthma, bacterial pneumonia, and lung opacity that can strongly correlate with COVID-19 will be considered to optimize the proposed algorithms. The proposed model can be easily extended to a multiclass classifier for discriminating COVID-19 from other pulmonary diseases mentioned above. Also, the variable morphology of airways and lung dimensions that can alter the diagnoses for different genders will be examined.

## Figures and Tables

**Algorithm 1:**
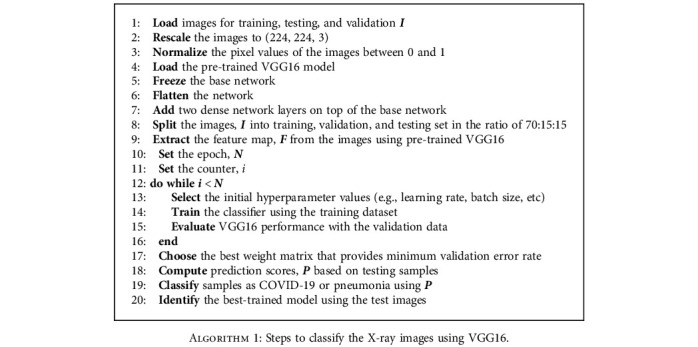
Steps to classify the X-ray images using VGG16.

**Algorithm 2:**
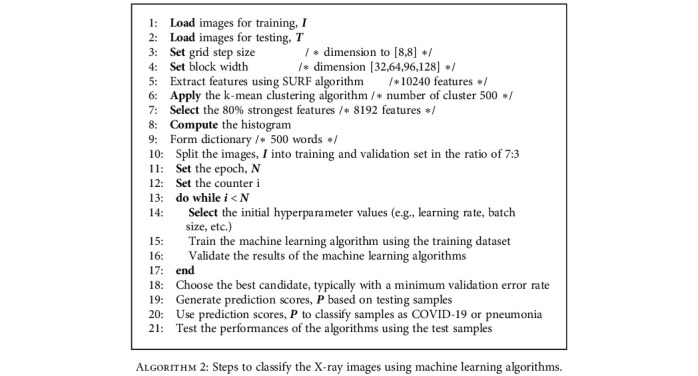
Steps to classify the X-ray images using machine learning algorithms.

**Table 1:** The list of acronyms and their definition.

Acronyms	Definition
AUC	Area under the curve
CLAHE	Contrast limited adaptive histogram equalization
CLD	Color layout descriptor
CNN	Convolutional neural network
CT	Computerized tomography
DCGAN	Deep convolutional generative adversarial network
DTL	Deep transfer learning
FDR	False detection rate
FN	False negative
FNR	False negative rate
FP	False positive
IoMT	Internet of Medical Things
kNN	*k*-nearest neighbors
LDA	Linear discriminant analysis
MADE	Multiobjective adaptive differential evolution
PCA	Principal component analysis
RSVM	Reduced support vector machine
PPV	Positive predictive value
ReLU	Rectified linear unit
RF	Random forest
ROI	Region of interest
RT-PCR	Reverse transcription-polymerase chain reaction
SARS-CoV-2	Severe acute respiratory syndrome coronavirus
SURF	Speeded-up robust feature
TN	True negative
TP	True positive
TPR	True positive rate
WHO	World Health Organization

**Table 2:** The best-trained model parameters for VGG16.

Hyperparameters	
Optimization method	RMSprop
Training mode	Auto
Patience (early stopping)	10
Dropout	25%
Batch size	10
Learning rate	0.0001
Epoch	30
Minimum detectable loss	0.00001
Learning reduction rate	0.1

**Table 3:** The performance of the system model with VGG16.

Pathology/performance measures	Precision	Recall	F1-score	FDR/FNR	*G*-mean	MCC
COVID-19	1.0	1.0	1.0	0	1.0	1.0
Pneumonia	1.0	1.0	1.0	0	1.0	1.0
Accuracy	1.0 1.0 1.0 0.0373					
Macroaverage						
Weighted average						
Log loss						

**Table 4:** The classification performance for COVID-19 with SURF features.

Classifier model	TPR/recall (%)	FNR (%)	PPV/precision (%)	FDR (%)	F1-score (%)
Fine tree	90.00	10.00	85.70	14.30	87.80
Linear discriminant	97.50	2.50	97.50	2.50	97.50
Logistic regression	97.50	2.50	97.50	2.50	97.50
Linear SVM	97.50	2.50	98.70	1.30	98.10
Coarse Gaussian SVM	98.80	1.20	97.50	2.50	98.15
Cosine kNN	78.80	21.30	98.40	1.60	87.51
Ensemble subspace discriminant	97.50	2.50	97.50	2.50	97.50

**Table 5:** The classification performance for pneumonia with SURF features.

Classifier model	TPR/recall (%)	FNR (%)	PPV/precision (%)	FDR (%)	F1-score (%)
Fine tree	85.00	15.0	89.50	10.70	87.20
Linear discriminant	97.50	2.50	97.50	2.50	97.50
Logistic regression	97.50	2.50	97.50	2.50	97.50
Linear SVM	98.80	1.20	97.50	2.50	98.14
Coarse Gaussian SVM	97.50	2.50	98.70	1.30	98.10
Cosine kNN	98.80	1.20	82.30	17.70	89.80
Ensemble subspace discriminant	97.50	2.50	97.50	2.50	97.50

**Table 6:** The performance comparison (machine learning models).

Classifier model	Accuracy (%)	Training rate (sec)	Prediction rate (ms/prediction)	AUC
Fine tree	87.5	09.72	3.22	0.88
Linear discriminant	97.5	10.20	3.33	0.99
Logistic regression	97.5	14.57	3.22	0.99
Linear SVM	98.1	11.07	2.70	0.99
Coarse Gaussian SVM	98.1	15.47	2.04	0.99
Cosine kNN	88.8	20.24	2.50	0.98
Ensemble subspace discriminant	97.5	20.24	5.26	0.99

**Table 7:** The performance comparison.

Study	Features	Classifier	Best Results/Findings
Hemdan [47]	Chest X-ray	VGG19, DenseNet	Accuracy: 90% (VGG19) Accuracy: 90% (DenseNet)
Arias-Garzón et al. [48]	Chest X-ray, lung segmentation	VGG19, U-Net	Accuracy: 97.05% (VGG19)
Bushra et al. [49]	Chest X-ray images	CNN	Accuracy: 98.65%, sensitivity: 98.49%, specificity: 98.82%, precision: 98.65%, and F1-score: 98.6%
Sharmila and Florinabel [50]	Chest X-ray	DCGAN-CNN	Accuracy: 98.6%
Brunese et al. [51]	Color layout descriptor	Machine learning	Precision: 0.965, recall: 0.965
Abugabah et al. [52]	Chest X-ray	COVID-3DS-CNN	Accuracy: 96.70%, specificity: 95.55%, and sensitivity: 96.62%
Apostolopoulos and Mpesiana [53]	Chest X-ray	VGG19 MobileNet-V2	Accuracy: 96.78%, sensitivity: 98.66%, and specificity: 96.46%
Manokaran et al. [54]	Chest X-ray	DNN	Accuracy: 94%
Madhavan et al. [55]	Chest X-ray	Res-CovNet	Accuracy: 98.4% (binary), 96.2% (multiclass)
Rahaman et al. [56]	Chest X-ray images	VGG16, VGG19, ResNet, DenseNet, MobileNet, Xception, and Inception	Highest accuracy: 89.3% (VGG19)
Albahli and Albattah [57]	Chest X-ray images	ResNet-V2, InceptionNet-V3, and NASNetLarge	Accuracy: 99.02% (InceptionNet)
Gouda et al. [58]	Chest X-ray images	ResNet-50	Accuracy: 99.63%, precision: 100%, recall: 98.89%, F1-score: 99.44, and AUC:100%
Awan et al. [59]	Chest X-ray images	Inception-V3 ResNet-50 VGG19	Highest accuracy: 100% (binary)
Mahesh et al. [60]	Chest X-ray images	CNN	Accuracy: 100%
Sarki et al. [61]	Chest X-ray images	CNN	Accuracy: 100% (binary), 93.75% (multiclass)
Ho and Gwak [62]	Handcrafted features Radiomic features Deep features	LDA, kNN, GNB, SVM, AdaBoost, RF, ensemble XGBoost, and NN	Accuracy: 89.2%, precision: 89.2%, recall: 89.2%, and F1-score: 89.2%
Rawat et al. [63]	Chest X-ray	Inception-V3, MobileNet, Xception, and DenseNet	Accuracy: 100% (InceptionV3)
Zouch et al. [64]	Chest X-ray CT scan	VGG19 and ResNet-50	Accuracy: 99.35% (VGG19), 96.77% (ResNet50)
Aggarwal et al. [65]	Contrast-limited adaptive histogram equalization (CLAHE)	Pretrained CNNs	Accuracy: 81%
Reshi et al. [66]	Chest X-ray	CNN	Accuracy: 99, precision: 1.0, sensitivity: 0.990, specificity: 1.0, F1-score: 0.994, and AUC: 0.990
Attaullah et al. [67]	Chest X-ray COVID-19 symptoms	Logistic regression and CNN	Accuracy: 78.88%, specificity: 94%, and sensitivity: 77%
Proposed method I	Chest X-ray images	VGG16	Accuracy: 100%, precision: 100%, recall: 100%, F1-score: 100%, G-mean: 1.0, MCC: 1.0, and AUC: 1.0
Proposed method II	SURF features	Machine learning algorithms	Accuracy: 98.1% linear/coarse Gaussian SVM) AUC: 0.99 (linear/course Gaussian SVM) Recall: 98.80% (linear SVM, cosine kNN) Precision: 98.70% (linear/coarse Gaussian SVM) F1-score: 98.15% (coarse Gaussian SVM)

## Data Availability

The data can be found in https://www.kaggle.com/tawsifurrahman/covid19-radiography-database.

## References

[1] Cucinotta D., Vanelli M. (2020). WHO declares COVID-19 a pandemic. *Acta Biomed*.

[2] Worldometer Corona Virus Cases. https://www.worldometers.info/coronavirus/.

[3] Bhatt T., Kumar V., Pande S., Malik R., Khamparia A., Gupta D., Al-Turjman F. (2021). A review on COVID-19. *Artificial Intelligence and Machine Learning for COVID-19 Studies in Computational Intelligence*.

[4] Nanda S. K., Ghai D., Pande S., Gupta D., Polkowski Z., Khanna A., Bhattacharyya S., Castillo O. (2022). VGG-16-based framework for identification of facemask using video forensics. *Proceedings of Data Analytics and Management Lecture Notes on Data Engineering and Communications Technologies*.

[5] Udugama B., Kadhiresan P., Kozlowski H. N. (2020). Diagnosing COVID-19: the disease and tools for detection. *American Chemical Society (ACS) Nano*.

[6] Mcneill K., Jacobs C. Half of world population lacks access to essential health services- are we doing enough. *World Economic Forum*.

[7] Islam R., Tarique M., Abdel-Raheem E. (2020). A survey on signal processing based pathological voice detection techniques. *IEEE Access*.

[8] Islam R., Tarique M. Classifier based early detection of pathological voice.

[9] Islam R., Tarique M. (2022). A novel convolutional neural network based dysphonic voice detection algorithm using chromagram. *International Journal of Electrical and Computer Engineering*.

[10] Lee L., Chamberlain L. G., Loudon R. G., Stemple J. C. (1988). Speech segment durations produced by healthy and asthmatic subjects. *Journal of Speech Hear Disorder*.

[11] Cummings J. L., Benson F., Hill M. A., Read S. (1985). Aphasia in dementia of the Alzheimer type. *Neurology*.

[12] Jankovic J. (2008). Parkinson's disease: clinical features and diagnosis. *Journal of Neurology, Neurosurgery, and Psychiatry*.

[13] Islam R., Abdel-Raheem E., Tarique M. (2022). Voice pathology detection using convolutional neural networks with electroglottographic (EGG) and speech signals. *Computer Methods and Programs in Biomedicine Update*.

[14] Nilsonne A., Sundberg J., Ternström S., Askenfelt A. (1988). Measuring the rate of change of voice fundamental frequency in fluent speech during mental depression. *Journal of Acoustic Society of America*.

[15] Elvevåg B., Foltz P. W., Rosenstein M., Delisi L. E. (2010). An automated method to analyze language use in patients with schizophrenia and their first-degree relatives. *Journal of Neurolinguistics*.

[16] Zhang J., Pan Z., Gui C., Zhu J., Cui D. (2016). Clinical investigation of speech signal features among patients with schizophrenia. *Shanghai Archives of Psychiatry*.

[17] Kanner L. (1946). Irrelevant and metaphorical language in early infantile autism. *The American Journal of Psychiatry*.

[18] Taib D., Tarique M., Islam R. Voice features analysis for early detection of voice disability in children.

[19] Islam R., Abdel-Raheem E., Tarique M. (2022). A novel pathological voice identification technique through simulated cochlear implant processing systems. *Applied Science*.

[20] Islam R., Tarique M. (2020). Blind source separation of fetal ECG using fast independent component analysis and principle component analysis. *International Journal of Scientific and Technology Research*.

[21] Graves K., Schmidt J. E., Bollmer J. (2005). Emotional expression and emotional recognition in breast cancer survivors: A controlled comparison. *Journal of Psychology and Health*.

[22] Islam R., Abdel-Raheem E., Tarique M. (2022). A study of using cough sounds and deep neural networks for the early detection of Covid-19. *Biomedical Engineering Advances*.

[23] Islam R., Abdel-Raheem E., Tarique M. Early detection of COVID-19 patients using chromagram features of cough sound recordings with a machine learning algorithm.

[24] Madhavan M. V., Thanh D. N. H., Khamparia A., Pande S., Malik R., Gupta D. (2021). Recognition and classification of pomegranate leaves diseases by image processing and machine learning techniques. *Computer Methods, Materials and Continua*.

[25] Yadav N., Alfayeed S. M., Khamparia A., Pandey B., Thanh D. N. H., Pande S. (2022). HSB model-based segmentation driven facial acne detection using deep learning. *Expert System*.

[26] Kundu R., Das R., Geem Z. W., Han G. T., Sarkar R. (2021). Pneumonia detection in chest X-ray images using an ensemble of deep learning model. *PLoS One*.

[27] Manickam A., Jiang J., Zhou Y., Sagar A., Soundrapandiyan R., Samuel R. D. J. (2021). Automated pneumonia detection on chest X-ray images: a deep learning approach with different optimizers and transfer learning architectures. *Measurement*.

[28] Hashmi M. F. (2021). Pneumonia detection in chest X-ray images using compound scaled deep learning model. *Journal for Control, Measurement, Electronics, Computing, and Communication*.

[29] Yao S., Chen Y., Tian X., Jiang R. (2021). Pneumonia detection using an improved algorithm based on faster R-CNN. *Computational and Mathematical Methods in Medicine*.

[30] Rahman T. (2020). Reliable tuberculosis detection using chest X-ray with deep learning, segmentation, and visualization. *IEEE Access*.

[31] Qin Z., Ahmed S., Sarker M. S. (2021). Tuberculosis detection from chest X-rays for triaging in a high tuberculosis- burden setting: an evaluation of five artificial intelligence algorithms. *Lancet Digital Health*.

[32] Duong L. T., le N. H., Tran T. B., Ngo V. M., Nguyen P. T. (2021). Detection of tuberculosis from chest X-ray images: boosting the performance with vision transformer and transfer learning. *Expert System with Applications*.

[33] Vizioli L., Ciccarese F., Forti P. (2016). Integrated use of lung ultrasound and chest X-ray in the detection of interstitial lung disease. *Respiration*.

[34] Bradley S. H., Hatton N. L. F., Aslam R. (2021). Estimating lung cancer risk from chest X-ray and symptoms: a prospective cohort study. *British Journal of General Practice*.

[35] Altorki N., Kent M., Pasmantier M. (2001). Detection of early-stage lung cancer: computed tomographic scan or chest radiograph?. *The Journal of Thoracic and Cardiovascular Surgery*.

[36] Rajagopalan K., Babu S. (2020). The detection of lung cancer using massive artificial neural network-based pon soft tissue technique. *BMC Medical Informatics and Decision Making*.

[37] Sowjanya M. N. (2016). Lung cancer detection in chest X-ray image. *International Journal of Research and Analytical Reviews*.

[38] Schultheiss M., Schmette P., Bodden J. (2021). Lung nodule detection in chest X-rays using synthetic ground-truth data comparing CNN-based diagnosis to human performance. *Scientific Reports*.

[39] Chen S., Han Y., Lin J., Zhao X., Kong P. (2020). Pulmonary nodule detection on chest radiographs using balanced convolutional neural network and classic candidate detection. *Artificial Intelligence in Medicine*.

[40] Oğul B. B., Koşucu P., Ízšam A., Kanik S. D., Lacković I., Vasic D. (2015). Lung nodule detection in X-ray images: a new feature set. *6th European Conference of the International Federation for Medical and Biological Engineering*.

[41] Homayounieh F., Digumarthy S., Ebrahimian S. (2021). An artificial intelligence-based chest X-ray model on human nodule detection accuracy from a multicenter study. *JAMA Network Open*.

[42] Mendoza J., Pedrini H. (2020). Detection and classification of lung nodules in chest X-ray images using deep convolutional neural networks. *An International Journal of Computational Intelligence*.

[43] Li X., Shen L., Xie X. (2020). Multi-resolution convolutional networks for chest X-ray radiograph based lung nodule detection. *Artificial Intelligence in Medicine*.

[44] Chung M., Bernheim A., Mei X. (2020). CT imaging features of 2019 novel coronavirus (2019-nCoV). *Radiology*.

[45] Chenxi L. (2020). A clinical study and CT findings of a familial cluster of pneumonia with coronavirus disease 2019 (COVID-19)]. *Journal of Sichuan University (Health Science)*.

[46] COVID-19 Radiography Database. https://www.kaggle.com/tawsifurrahman/covid19-radiography-database.

[47] Hemdan E. E.-D. Covidx-net: a framework of deep learning classifiers to diagnose covid-19 in X-ray images. https://arxiv.org/abs/2003.11055.

[48] Arias-Garzón D., Alzate-Grisales J. A., Orozco-Arias S. (2021). COVID-19 detection in X-ray images using convolutional neural networks. *Machine Learning with Applications*.

[49] Bushra K. F., Ahamed M. A., Ahmad M. (2021). Automated detection of COVID-19 from X-ray images using CNN and Android mobile. *Research on Biomedical Engineering*.

[50] Sharmila V. J., Florinabel J. (2021). Deep learning algorithm for COVID-19 classification using chest X-ray images. *Computational and Mathematical Methods in Medicine*.

[51] Brunese L., Martinelli F., Mercaldo F., Santone A. (2020). Machine learning for coronavirus covid-19 detection from chest X-rays. *Procedia Computer Science*.

[52] Abugabah A., Mehmood A., Zubi A. A. A. L., Sanzogni L. (2022). Smart COVID-3D-SCNN: a novel method to classify X-ray images of COVID-19. *Computer Systems Science and Engineering*.

[53] Apostolopoulos I. D., Mpesiana T. A. (2020). Covid-19: automatic detection from X-ray images utilizing transfer learning with convolutional neural networks. *Physical and Engineering Sciences in Medicine*.

[54] Manokaran J., Zabihollahy F., Hamilton-Wright A., Ukwatta E. (2021). Detection of COVID-19 from chest x-ray images using transfer learning. *Journal of Medical Imaging*.

[55] Madhavan M. V., Khamparia A., Gupta D., Pande S., Tiwari P., Hossain M. S. (2021). Res-CovNet: an internet of medical health things driven COVID-19 framework using transfer learning. *Neural Computing and Applications*.

[56] Rahaman M. M., Li C., Yao Y. (2020). Identification of COVID-19 samples from chest X-ray images using deep learning: a comparison of transfer learning approaches. *Journal of X-ray Science Technology*.

[57] Albahli S., Albattah W. (2020). Detection of coronavirus disease from X-ray images using deep learning and transfer learning algorithms. *Journal of X-ray Science Technology*.

[58] Gouda W., Almurafeh M., Humayun M., Jhanjhi N. Z. (2022). Detection of COVID-19 based on chest X-rays using deep learning. *Healthcare*.

[59] Awan M. J., Bilal M. H., Yasin A., Nobanee H., Khan N. S., Zain A. M. (2021). Detection of COVID-19 in chest X-ray images: a big data enabled deep learning approach. *International Journal of Environmental Research and Public Health*.

[60] Mahesh P., Prathyusha Y. G., Sahithi B., Nagendram S. COVID-19 detection from chest X-ray using convolutional neural network.

[61] Sarki P., Ahmed K., Wang H., Zhang Y., Wang K. (2022). Automated detection of COVID-19 through convolutional neural network using chest X-ray images. *PLoS One*.

[62] Ho T. K. K., Gwak J. (2022). Feature level ensemble approach for COVID-19 detection using chest X-ray images. *PLoS One*.

[63] Rawat R. M., Garg S., Jain N., Gupta G. COVID-19 detection using convolutional neural network architectures based upon chest X-ray images.

[64] Zouch W., Sagga D., Echtioui A. (2022). Detection of COVID-19 from CT and chest X-ray images using deep learning models. *Annals of Biomedical Engineering*.

[65] Aggarwal S., Gupta S., Alhudhaif A., Koundal D., Gupta R., Polat K. (2021). Automated COVID-19 detection in chest X-ray images using fine-tuned deep learning architectures. *Expert Systems*.

[66] Reshi A. A., Rustam F., Mehmood A. (2021). An efficient CNN model for COVID-19 disease detection based on X-ray image classification. *Complexity*.

[67] Attaullah M., Ali M., Almufareh M. F. (2022). Initial stage COVID-19 detection system based on patients’ symptoms and chest X-ray images. *Applied Artificial Intelligence*.

[68] Sekeroglu B., Uzsahin I. (2020). Detection of COVID-19 from chest X-ray images using convolutional neural networks. *SLAS Technology*.

[69] Sing D. (2021). Deep neural network-based screening model for COVID-19 infected patients using chest X-ray images. *International Journal of Pattern Recognition and Artificial Intelligence*.

[70] Simonyan K., Zisserman A. Very deep convolutional networks for large scale image recognition.

[71] Google Colaboratory Lab online. https://colab.research.google.com/.

[72] Loussaief S., Abdelkrim A. Deep learning vs bag of features in machine learning for image classifications.

[73] Bay H., Ess A., Tuytelaars T., van Gool L. (2008). Speeded-up robust features (SURF). *Computer Vision and Understanding*.

[74] An S., Ma X., Song R., Li Y. Face detection and recognition with SURF for human-robot interaction.

[75] Anand B., Shah P. K. (2016). Face recognition using SURF features and SVM classifier. *International Journal of Electronics Engineering Research*.

[76] Carro R. C., Larios J.-M. A., Huerta E. B., Caporal R. M., Cruz F. R. Face recognition using SURF.

[77] Segundo M. P., Gomes L., Bellon O. R. P., Silva L. Automating 3D reconstruction pipeline by surf-based alignment.

[78] Du G., Su F., Cai A. Face recognition using SURF features.

[79] Virginás-Tar Á. Face recognition using SURF interest points. http://www.scribd.com/doc/145823477/%20Face-Recognition-Using-SURFInterest-Points.

[80] Lim Y., Lee C. Robust inlier feature tracking method for multiple pedestrian tracking.

[81] Rangayyan R. M. (2015). *Biomedical Signal Analysis*.

[82] Jiaa Y., Du P. (2016). Performance measures in evaluating machine learning-based bioinformatics predictors for classifications. *Quantitative Biology*.

